# Pulmonary Co-infection with *Cryptococcus* Species and *Pneumocystis jirovecii* in an Old Patient Without a Previous Predisposing Illness

**DOI:** 10.1007/s11046-022-00651-8

**Published:** 2022-08-08

**Authors:** Jinbao Huang, Heng Weng, Changqing Lan, Hongyan Li

**Affiliations:** 1 Department of Respiratory Medicine, People’s Hospital Affiliated to Fujian University of Traditional Chinese Medicine, Fuzhou, 350004 China; 2 Department of Radiology, Fuzhou Pulmonary Hospital of Fujian, Educational Hospital of Fujian Medical University, Fuzhou, 350008 China; 3 Department of Critical Care Medicine, People’s Hospital Affiliated to Fujian University of Traditional Chinese Medicine, No. 602 Middle 817 Road, Taijiang District, Fuzhou, 350004 China

**Keywords:** *Pneumocystis jirovecii*, *Pneumocystis jirovecii* pneumonia, Cryptococcosis

## Abstract

**Background:**

Cryptococcosis and pneumocystosis are opportunistic infections which are more common in immunosuppressed individuals. Herein, we report a rare case of coinfection of pulmonary cryptococcosis (PC) and *Pneumocystis jirovecii* pneumonia (PJP) in a patient without a previous predisposing illness.

*Case presentation* A 76-year-old man was admitted to our hospital with complaints of cough, expectoration, shortness of breath, and fever. A chest computed tomography (CT) showed multiple nodules with diffuse ground-glass opacities (GGOs) in both lungs. The patient was diagnosed as extrinsic allergic alveolitis (Pigeon breeder’s lung). After treatment with corticosteroids, the patient improved with significant absorption of GGOs on chest CT. However, pulmonary nodules gradually enlarged and such lesions could not be explained by EAA. Based on the positivity of serum cryptococcal antigen and pathological examination of lung nodule which confirmed the presence of *Cryptococcus* spores, PC was diagnosed later and fluconazole was administered. However, repeated chest CT performed about 2 months after antifungal treatment showed significantly increased GGOs in both lungs. The pathological examination of new lung lesions revealed the presence of *P. jirovecii*. The patient was finally diagnosed having coinfection of PC and PJP and sulfamethoxazole was further prescribed. Thereafter, the patient improved again with significant absorption of GGOs as noted on chest CT.

**Conclusions:**

Concomitant PC and PJP is very rare, especially in a patient without a previous predisposing illness. Additionally, when pulmonary lesions cannot be completely explained by one kind of infectious disease, the possibility of mixed infection should be considered.

A 76-year-old man with cough, expectoration, shortness of breath and fever was admitted to our hospital. The patient had no history of any underlying disease. He had been fond of raising pigeons for years. A chest computed tomography (CT) showed multiple nodules with diffuse ground-glass opacities (GGOs) in both lungs (Fig. 1). A decrease in peripheral blood total T lymphocytes (CD3+) and their subtypes (CD4+, CD8+) was noted. CT-guided percutaneous needle biopsy (PNB) of nodule in the left lower lung (Fig. 1, red arrow) was performed, and the pathological examination revealed chronic inflammatory changes with large areas of necrosis. Various special stains were all negative, leading to an indefinite diagnosis. Althouth the patient received anti-infective therapy with moxifloxacin and ganciciovir, his condition continuously deteriorated. Since the patient refused tracheoscopy, bronchoalveolar lavage fluid (BALF) and transbronchial lung biopsy (TBLB) specimens were not available for further diagnosis at that time. Based on diffuse GGOs on chest imaging, a history of pigeon breeding, and the failure of conventional anti-infective therapy, the patient was clinically diagnosed as extrinsic allergic alveolitis (EAA, Pigeon breeder's lung). The empirical anti-inflammatory treatment with methylprednisolone (glucocorticoid) 40 mg once daily was initiated. Two weeks after therapy, repeated chest CT showed significant absorption of GGOs in both lungs. Thereafter, glucocorticoid was discontinued.

**Fig. 1:**
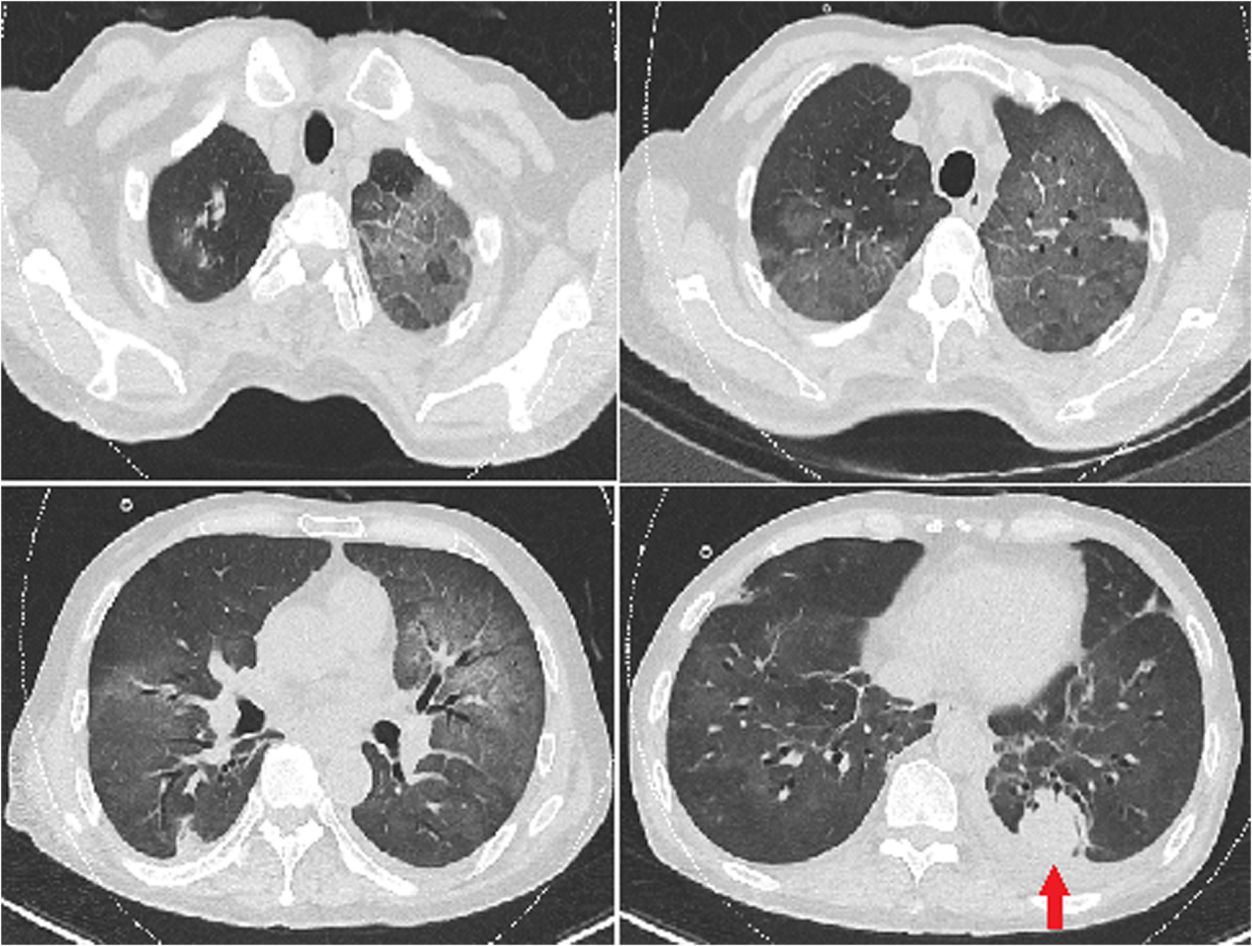
Chest CT showing multiple nodules with diffuse ground-glass opacities were observed in both lungs. First percutaneous needle biopsy of nodule was performed in the left lower lung (red arrow)

Since pulmonary nodules gradually enlarged and such lesions could not be explained by EAA, an additional detection of serum cryptococcal antigen was performed and the result was positive. Meanwhile, PNB of enlarging nodule in the right upper lung was performed (Fig. 2a, red arrow), and the pathological examination revealed the presence of *Cryptococcus* spores (Fig. 2b). Lumbar puncture was performed and the results of cerebrospinal fluid testing were normal. The patient was diagnosed as pulmonary cryptococcosis (PC) with multiple nodules and fluconazole 400 mg once daily was initiated. After treatment, chest CT images showed increase in size of nodules in bilateral lung. However, 2 months after antifungal treatment, the patient aggravated again. Repeated chest CT showed new GGOs significantly increased in both lungs (Fig. 3). TBLB was performed in the right upper lung where GGOs significantly progressed (Fig. 3, red arrow). Routine culture of BALF was negative; however, direct Periodic acid-Schiff stain and Gomori's methenamine silver stain on biopsy samples were positive for *Pneumocystis jirovecii* (Fig. 4)*.* The patient was considered to have coinfection of PC (nodular lesions) and *P. jirovecii* pneumonia (PJP, GGOs lesions). He was treated with additional compound sulfamethoxazole. Dynamic monitoring chest CT images showed that the lesions including nodules and GGOs continuously absorbed. The patient remained well without signs of recurrence 5 months after onset. Unfortunately, due to the outbreak of coronavirus disease 2019 (COVID-19) pandemic at the end of 2019, the patient was lost to follow-up at last.

**Fig. 2:**
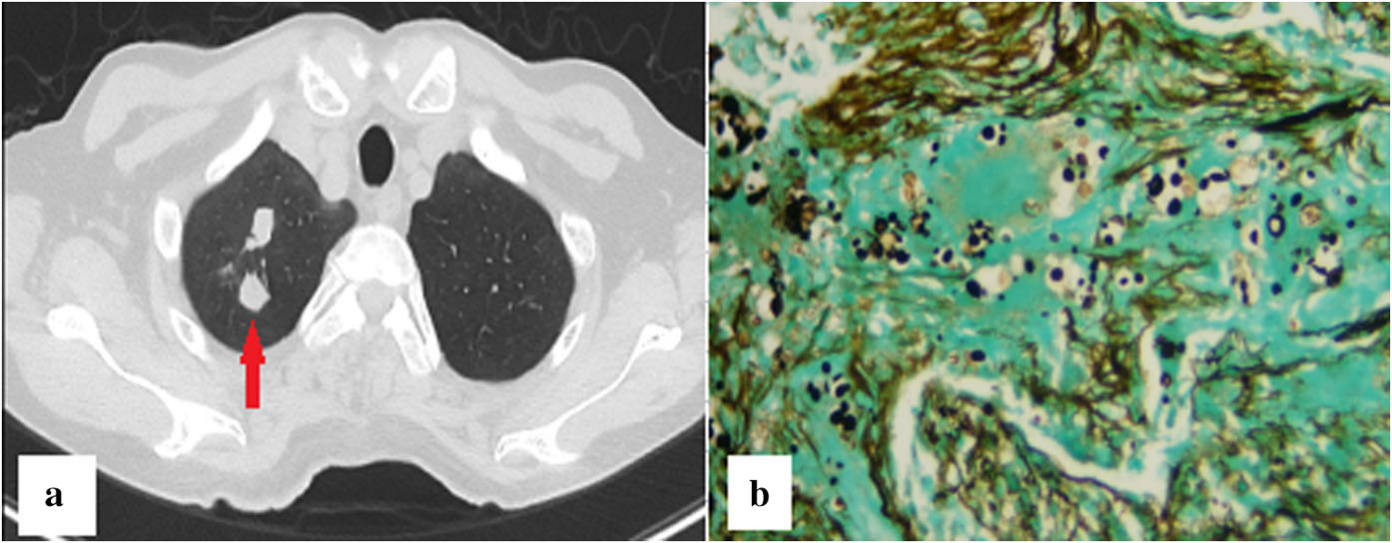
**a**, **b** Image of pathological examination following second percutaneous needle biopsy of enlarged nodule in the right upper lung (a, red arrow) depicting the presence of *Cryptococcus* spores throughout the granulation tissue on Gomori's methenamine silver stain (**b**).

**Fig. 3:**
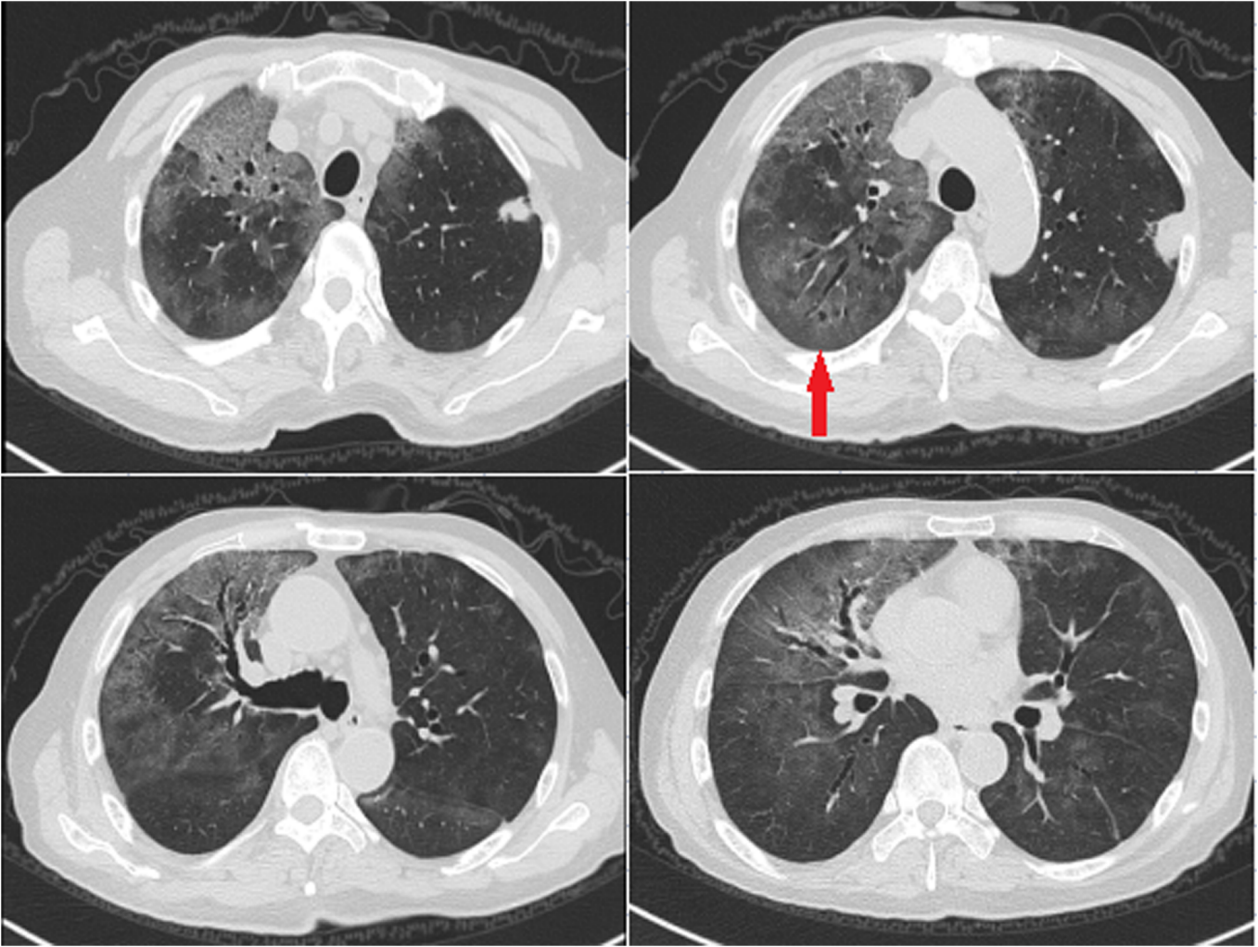
Repeated chest CT showing persistently progressed ground-glass opacities in bilateral lungs. Transbronchial lung biopsy was performed in the right upper lung (red arrow)

**Fig. 4:**
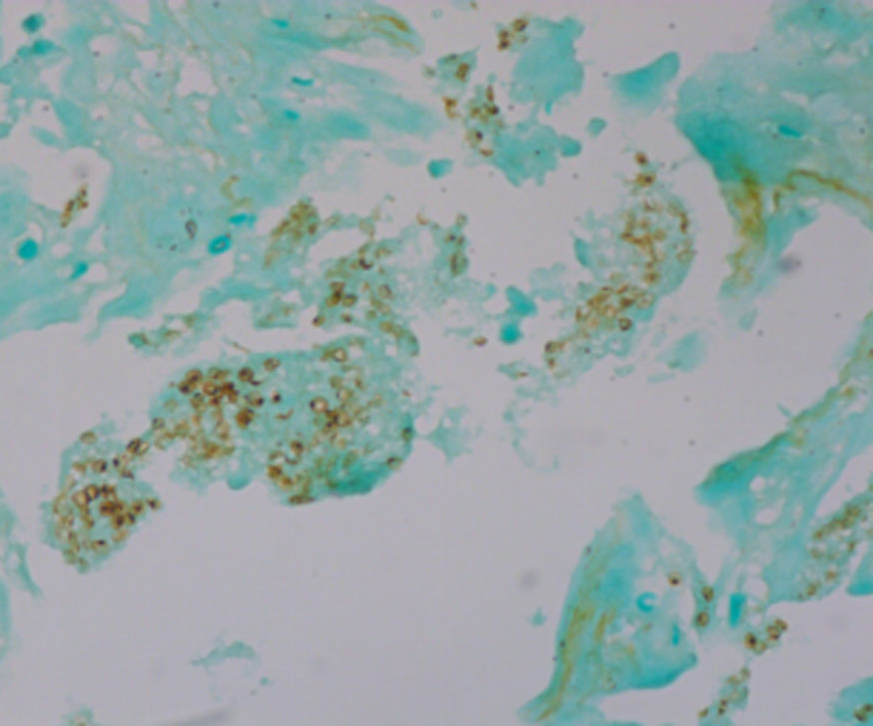
Gomori's methenamine silver stain of the right upper lung biopsy tissue showing *Pneumocystis* organisms

Concomitant PC and PJP is very rare, and mainly reported in HIV-infected patients and other immunosuppressed hosts. Surprisingly, this patient did not have definite immunosuppressed underlying diseases. It was considered that PC (nodular lesions) combined with EAA (first appearance of GGOs lesions on admission, Fig. 1) which was induced by allergens from pigeons appeared at first; and secondary PJP (second appearance of GGOs lesions at the late course, Fig. 3) appeared 2 months after anti-cryptococcus treatment. The long-term history of pigeon breeding which might have been associated with PC since pigeon dung has been documented as an important source of infection with *Cryptococcus*. PJP was probably secondary to corticosteroid therapy in the patient with prealable pneumonia and low lymphocytes count. However, two particular infections were detected in this patient without known predisposing illness, it was speculated that the patient may have an undetected potential immunodeficiency disease. Due to the limitations of our hospital in undertaking detailed laboratory investigations and the patient's financial constraints, the further screening of potential pre-existing systemic immunodeficiency diseases could not be performed. Furthermore, as seen in our case, when pulmonary lesions cannot be completely explained by one kind of infectious disease, the possibility of mixed infection should be considered.

